# Smartphone Time Machine: Tech-Supported Improvements in Time Perspective and Wellbeing Measures

**DOI:** 10.3389/fpsyg.2021.744209

**Published:** 2021-11-03

**Authors:** Julia Mossbridge, Khari Johnson, Polly Washburn, Amber Williams, Michael Sapiro

**Affiliations:** ^1^TILT: The Institute for Love and Time, Sebastopol, CA, United States; ^2^University of San Diego, San Diego, CA, United States; ^3^Institute of Noetic Sciences, Petaluma, CA, United States

**Keywords:** time perspective, wellbeing, transcendence technology, adverse childhood experiences, mental time travel, hope, unconditional love, prospection

## Abstract

Individuals with a balanced time perspective, which includes good thoughts about the past, awareness of present constraints and adaptive planning for a positive future, are more likely to report optimal wellbeing. However, people who have had traumas such as adverse childhood experiences (ACEs) are likely to have less balanced time perspectives and lower overall wellbeing when compared to those with fewer or no ACEs. Time perspective can be improved via *time-travel narratives* that support people in feeling connected to a wise and loving future version of themselves, an approach that has until now only been provided in counseling contexts. Our team used an iterative inclusive design process to shape a scalable time-travel narrative tool – a responsive and progressive web application called *Time Machine.* Among other functionalities, Time Machine allowed people to record and listen to messages as if they were from and to their past and future selves. Using pre-planned as well as *post-hoc* analyses, we analyzed quantitative and qualitative data from 96 paid design partners (participants) who were taken through a 26-day pilot study of the technology. Among other effects, the results revealed: (1) high engagement throughout the design process, (2) improvements in self-reported time perspective and overall wellbeing scores that were greater for those using Time Machine during an optional-use period, (3) twice as much improvement in overall wellbeing scores for design partners with high ACEs (16%) versus low ACEs (8%), and (4) feelings of unconditional love apparently mediating the relationship between scores on time perspective and overall wellbeing measures. We discuss the limitations of these results as well as implications for the future role of spiritually informed scalable time-travel narrative technologies in healthcare and wellness.

## Introduction

The ideal time perspective has been a topic of popular discussion for centuries. For instance, two forms of time were personified by the Greeks: Kronos vs. Kairos; these are similar in meaning to two Hindu words for time, Kala vs. Ritu (e.g., Makridakis, 2012; Lindley et al., 2013; Valentine, 2020). According to these accounts, Kronos/kala both refer to the externally measured chronological aspect of events, while Kairos/ritu both refer to the internally measured, subjectively experienced aspect of events. Meanwhile in recent academic philosophy, an argument has emerged in which some philosophers state that a fully rational human would have no temporal bias; for instance, a rational human would have no preference to have the bad things in life behind us and the good things ahead of us, because we are always the same person having the experience (Sullivan, 2018). Another philosophical camp states that it is both reasonable and common to have temporal biases such as these, or in any case that such temporal biases can be rationalized (Greene et al., 2021).

Beyond these philosophical speculations, recent interest in the impact of time perspective on human wellbeing has been rekindled by empirical investigations motivated by the recognition that subjective wellbeing and positive affect are related to one’s relationship to the temporal features of one’s own life (Zimbardo and Boyd, 1999; Boniwell et al., 2010; Zhang et al., 2013a; Stolarski, 2016; Cordonnier et al., 2018; Salgado and Berntsen, 2018; Sanson et al., 2018; Jankowski et al., 2020). For instance, “mental time travel” can be used to prospectively “pre-live” events based on templates from past experiences, and in fact such future-focused mental time travel has been found to be generally more positive and evocative than mentally visiting past events to direct behavior (Sanson et al., 2018). This could be due to a positivity bias when it comes to near-term future prospection (Salgado and Berntsen, 2018), which may arise because memories of actual events constrain our re-representations of the past, but not the future (Cordonnier et al., 2018). What has been called a “balanced time perspective” represents an intricate interplay between realistic and positive awareness of the past and present combined with willingness to hope and plan for a realistically positive future. Those with more positive and less negative thoughts about the past, non-fatalistic thoughts about the present, and a greater frequency of future-engaged thoughts are more likely to rank themselves as optimally functioning, having greater wellbeing, experiencing less negative affect and more life satisfaction than those who do not share this balanced time perspective (for review, see Jankowski et al., 2020).

For depressed adolescents and adults, realistic optimism and strong future orientation have been associated with decreased depressive and suicidal symptoms (Puskar et al., 1999; Gillham and Reivich, 2004; Hirsch et al., 2006, 2007, 2009; Chang et al., 2019). Among under-resourced youth, violence decreases as future orientation improves over time (Stoddard et al., 2011), robust future orientation has been considered a protective factor in high-violence communities (So et al., 2018), and among abused youth, future orientation improves as resources improve (Oshri et al., 2018). Further, future time perspective predicted the success of inmates in a vocational training program (Chubick et al., 1999). But a balanced time perspective is not isolated to the future. For instance, research on daydreaming indicates that dreams of a very positive future that do not match at all our present circumstances — as in “I’m sick with cancer now but tomorrow I’ll be well,” — actually increase depression, even though the fantasies seem to induce some happiness in us (Oettingen et al., 2016).

Not surprisingly, our time perspectives are informed by our experiences. For example, in one study, people with post-traumatic stress disorder from a car accident experience had levels of PTSD that were partially remediated by having a more balanced time perspective (Stolarski and Cyniak-Cieciura, 2016). Further, in an examination of lifetime exposure to trauma and optimism for the future, deviations from a balanced time perspective partially mediated the relationship between more trauma exposure and less optimism (Tomich and Tolich, 2021). The broader implication of such studies is that those of us who have experienced trauma but have balanced time perspectives may be more likely to report higher overall wellbeing than those who have experienced trauma but have a time perspective that deviates greatly from optimal balance. Thus interventions that move people toward a balanced time perspective could be beneficial, especially for those who have experienced trauma.

Time perspective does seem amenable to intervention, sometimes leading to increases in wellbeing or other positive correlates of a balanced time perspective. Research in positive psychology has shown that a narrative approach can be an empowering tool for positive change (White and Epston, 1990; Zimmerman and Dickerson, 1996; Sheldon and Vansteenkiste, 2006). Some practitioners and researchers have used what we call “time-travel narratives,” in which people are coached to use ideas about past and future versions of themselves to create more balanced time perspectives and achieve positive behavioral changes, both within therapeutic contexts (Newsome, 2004; Kress et al., 2008, 2011; Hoffman et al., 2010; Palgi and Ben-Ezra, 2010; Madden et al., 2011; Palgi et al., 2014) and outside of them (Hall and Fong, 2003; Schuitema et al., 2014; Snider et al., 2016). However, research on the efficacy of time travel narratives as an independent intervention not accompanied by other coaching or therapies is in its infancy, probably because it is expensive to target in-person interventions to a large number of appropriate individuals.

Following the recent trend of making evidence-based positive psychology tools available via scalable mobile and internet applications (Mossbridge, 2016; Diefenbach, 2018; Kitson et al., 2018; Miller and Polson, 2019), we set out to create an affordable, accessible, evidence-based, self-administered, time-travel narrative technology designed to balance time perspectives and potentially improve wellbeing. We define a time-travel narrative technology as any scalable tool that helps people build the habit of working to heal negative memories of the past and weaving an engaged present into their hopes and goals for a positive future. We were especially focused on co-designing the technology with those who had experienced trauma, including abuse, addiction, poverty, incarceration, and neglect. This is because in our previous experience with evidence-based technology development, we had become acquainted with the principle that when technology is not designed by the intended beneficiaries, the technology ends up not being beneficial (McGuinness and Schank, 2021). Thus we had two aims for this pilot study: (1) create an inclusive design process to support the creation of a prototype time-travel narrative tool, and (2) test the efficacy of this prototype. Our primary research questions were whether our inclusively designed prototype could support people in balancing their time perspectives, increasing feelings of unconditional love [as defined in Mossbridge et al. (2021); see Methods “Recurring Assessments (Study Days 2, 8, 14, 25)”], and improving measures of physical and overall wellbeing. Here we offer an overview of the inclusive design process as well as quantitative and qualitative results that shed light on these questions.

## Materials and Methods

### Procedure

#### Focus Groups and Demographics Survey Completion

To reach our first aim of creating an inclusive software design and testing process, we invited all screened and consented participants to a ∼1.5-hour online (Zoom) focus group. The first and last authors (JM and MS) facilitated each focus group. During the first 30 min of each focus group, the first author briefed the participants on the scientific background, the overall study design, and their important role as design partners. Then we gave the design partners a link to an online demographics survey and the Adverse Childhood Experiences Survey (ACES; Felitti et al., 1998). After these surveys, the last author led the design partners in a 15–20 min time travel narrative meditation, in which they were encouraged to imagine visiting a past and a future version of themselves, and were especially encouraged to imagine being loved by their future self. After this meditation, both facilitators shared how time travel narratives like this one have helped them. Finally, we opened up the meeting for a 20- to 30-min period during which design partners debriefed from their experiences and asked questions about the technology and the study. During the entire focus group, we supported the sharing of private information only when design partners left their video and audio feeds off, created anonymous screen names, and knew how to privately communicate via the chat window to the two facilitators. When the debriefing seemed complete and all questions were answered, our design partners were reminded of the payment schedule, the fact that they could drop out at any time and receive prorated payment for the work they had already done, and the fact that they could take as long as they like (up to 5 months) to complete the 26 days of the study.

#### Technology Development

**FIGURE 1:**
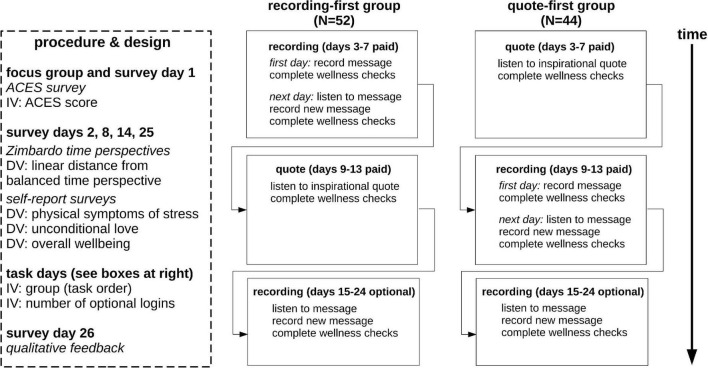
Schematic of study design. We used a crossover design comparing two task orders (recording-first, quote-first). All 96 design partners engaged in all required (paid) days, spread over as few as 26 days (shortest time frame) to 5 months (longest time frame). ACES = Adverse childhood experiences study.

Our second aim was to design, build and test accessible and scalable software to engage our population in a time travel narrative task (“recording task”) and compare the benefits of engaging in that task, if any, with benefits derived from a control task (“quote task”; Figure 1 and below). We chose a browser-based progressive web application coded in JavaScript, CSS and HTML 5+ with a Firebase backend; we decided not to create a pre-packaged mobile app so the technology could eventually become available to incarcerated people who are more likely to have access to the internet without access to mobile apps. Our design, development, and project management leads (AW, KJ, and PW, respectively) created and executed an iterative roll-out process that allowed us to continually update design and functionality throughout the study without altering any of the primary scientifically important aspects of the technology. This allowed us to respond to feedback from design partners about features they would like to see in future iterations of the technology. This “Time Machine” technology used a “dashboard” approach to present four components to our design partners: efficacy tracking, the recording task, the quote task, and wellness checks. All three were timed as “to-do” items on the dashboard, and all followed the study schedule. Design partners could skip days and put the study on hold for up to 4 months, but the technology required them to complete all 10 days of the experimental and control conditions and all required surveys if they chose to remain in the study. Each component was presented to the user at the required time, according to the study timeline (Figure 1). Each of these components are described below.

#### Efficacy Tracking

To measure the efficacy of the technology, we used a between-groups crossover design. The Time Machine software randomly assigned design partners to one of two groups: recording-first group (*N* = 52) or quote-first group (*N* = 44; Figure 1). The experimental condition was the recording task (see below) and the control condition was the quote task (see below). The four dependent variables were the linear deviation from a balanced time perspective (see section “Data Analysis”) and responses to questions related to physical symptoms of stress, feelings of unconditional love, and overall wellbeing (see Surveys). These variables were calculated from responses to each of the four recurring assessment surveys administered on days 2, 8, 14, and 25 (see surveys, below), which were administered on a HIPAA-enabled survey site (FormSite) launched from the dashboard of the Time Machine. The independent variables were the ACES score taken from the survey administered during the focus group (day 1; see Surveys), group (task order), and the number of logins to the Time Machine during the 10-day unpaid optional period of the study. To ensure private and accurate storage of information pertinent to each design partner, FormSite received a hashed version of the design partner’s identity via the URL used to launch the survey site, and passed back to Time Machine a confirmation that the design partner had completed a survey. However, no survey responses were stored within the Firebase database.

#### Recording Task

The recording task was a self-guided scalable replication of a time-travel narrative. Design partners were asked to record a message to their past and future selves, and then send the message into their time machine. The next day, design partners were given access to the previous day’s message from the time machine and asked to listen to their message with love for themselves. They were not instructed how to do this, but were asked to try to imagine doing so. The aim of the recording task was to help give our design partners a daily reminder that they are continuously existing entities, that they are getting through each day and moving onto the next, and that they can develop a positive relationship to their internal representations of their future selves. During the focus group, this task was described as the task that was most like the guided meditation conducted during the focus group, so our design partners were aware that this task was of most interest to us (i.e., they were not blind to the study design). After the first recording was made, no advancement in the study was allowed without playing the previous day’s recording and pressing a button stating “I’m Done.”

#### Quote Task

In the quote task, design partners were encouraged to listen with love to randomly selected recording of one of our project staff reading an inspirational quote. The quote being read was also shown on the screen. This task was meant to be as close to the recording task as possible without presenting the design partner with their own voice and without relating to past or future selves. In this way, it controlled for the act of being encouraged to listen to positive words with love. No advancement in the study was allowed without playing the recording and pressing a button stating “I’m Done.”

#### Experimental Wellness Checks

**FIGURE 2:**
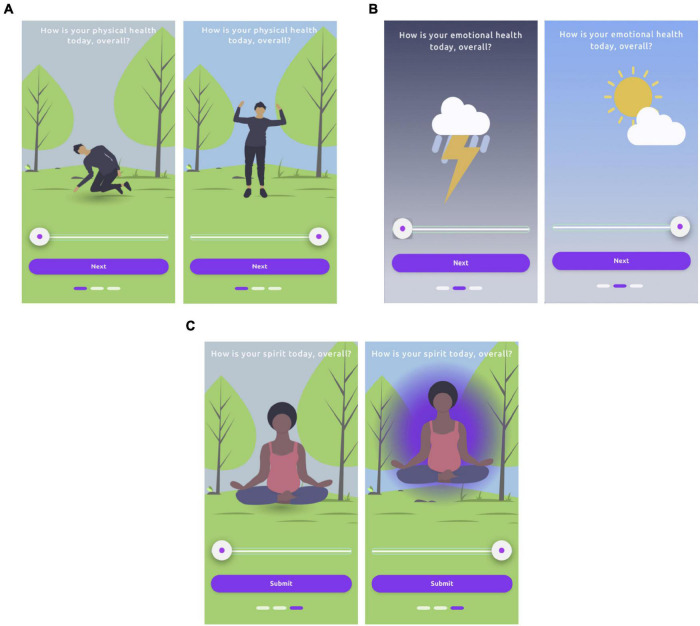
Wellness check sliders showing discoverable animations indicating meaning of slider positions; left = low rating and right = high rating in all cases. **(A)** physical, **(B)** emotional, **(C)** spiritual. Three rectangles at the bottom of the screen indicate slider progress. All three sliders were required to be moved before a task day (or non-survey day) was considered complete; default position was center. We analyzed data from these wellness checks, though they were not among the formally defined dependent variables.

Wellness checks consisted of three animated sliders presented in this order: physical, emotional, and spiritual wellness. To boost engagement, we allowed design partners to discover that moving the sliders to the right (highest score: 10) produced animated positive changes in the graphics for each slider, while moving the sliders to the left (lowest score: 0) produced animated negative changes (Figure 2). These wellness checks were presented right after design partners completed their recording or quote task on any non-survey day. No advancement in the study was allowed without completing the wellness checks for that day; the default position for each slider was in the middle of the screen. Because of the experimental nature of the wellness checks, responses were not included as part of the formal set of dependent variables, but we present analyses of the data they produced regardless (see section “Results”).

### Hypotheses

We tested five hypotheses in this pilot study. All tests of these hypotheses were pre-planned.

Hypothesis 1. Measures of time perspective, physical symptoms of stress, unconditional love, and overall wellbeing (i.e., all dependent variables) will improve from the first to last days of the study for participants who use the Time Machine technology for the required study period.

Hypothesis 2. Individuals who perform the recording task prior to the quote task will show greater improvement from the first to last days of the study in all dependent variables than participants who perform the tasks in the reverse order.

Hypothesis 3. All dependent variables will initially be better (more adaptive) for individuals who report relatively lower versus higher numbers of ACES.

Hypothesis 4. Individuals with lower numbers of ACES will report greater improvement from the first to last days of the study in all dependent variables than individuals with higher numbers of ACES.

Hypothesis 5. Improvements from the first to last days of the study in all dependent variables will be greater with greater use of the technology during the optional study period.

### Design Partners (Participants)

All design partners were asked to read and sign an online consent form. No design partner was invited to the online focus group or given access to the time travel narrative technology unless they consented. Human subjects protocols and consent processes were approved by the Institute of Noetic Sciences Institutional Review Board under approval number MOSJ_2020_01. From August 1, 2020-Dec 15, 2020 we enrolled 104 design partners in the study. Nineteen were recruited as a result of social workers and addiction counselors distributing our flier, the remaining were pre-screened with a survey advertised on Amazon Mechanical Turk (mTurk) and Turker Nation, the Slack community for “Turkers” (workers on mTurk). The pre-screening included all the demographics and ACEs questionnaire information that was also requested on day 1 of the study (see below). The pre-screening screened out non-native English speakers as well as participants who reported neither adverse childhood experiences nor belonging to any traditionally under-resourced group (non-white, LGBTQ+, disabilities, incarceration, United States military service). All participants were over age 18, but beyond that cutoff requirement no further age information was requested from participants. Participants in the pilot study participated in 3–4 total engagement hours over 26 days, with 16 days in which engagement was required. The remaining 10 engagement days were optional. Each design partner who finished the entire experiment received $180. No design partner chose to withdraw from the study and receive prorated payment.

### Efficacy Tracking Surveys

Figure 1 gives a schematized version of the efficacy tracking timeline and study design; here we briefly describe each survey.

#### Focus Group and Demographics (Study Day 1)

On the first study day, design partners attended the 90-min focus group, completed a modified ACES inventory {described in Felitti et al. (1998), validated in Murphy et al. (2014) [Chronbach’s alpha = 0.88]} with non-required responses asking permission to ask questions about a particular form of abuse prior to providing those questions, as well as: “What is your race/ethnicity?” (open text field), “What is your gender?” (open text field), “Do you identify as LGBTQ+?” (yes/no), “Do you have disabilities that have affected your life?” (yes/no), “Do you have a history of incarceration?” (yes/no/somewhere in between [“somewhere in between” scored as yes]), “Have you ever served in the United States military?” (yes/no).

#### Recurring Assessments (Study Days 2, 8, 14, 25)

The recurring assessment was performed four times during the course of the study, and contained four measures:

(1) The brief Zimbardo time perspective inventory [as described and validated in Zhang et al. (2013b); test-retest validity 0.73, mean 0.78 correlation with longer Zimbardo measure]. Scores on this measure were used to calculate the deviation from a balanced time perspective (see Data Analysis, below). In the present study, test-retest validity averaged 0.70.
(2) A physical symptoms of stress scale with time frame over the past 3 days [as used and described in Lee et al. (2010) and originally developed by Patchen (1970) but not validated]. As in Lee et al. (2010), physical symptoms of stress were: headache, upset stomach, gas or bloated feeling, and trouble getting to sleep. Participants used a 4-part scale to rate the occurrences of each symptom (0, 1, 2, or 3+ times), and the sum of the occurrences (with 3 points for 3+ times) was used as the score. In the present study, test-retest validity averaged 0.67.
(3) A feelings of unconditional love scale with time frame over the past 3 days [as used and validated in Mossbridge et al. (2021); test-retest validity mean of 0.87, correlation with unvalidated love questionnaire mean of 0.67]. The definition of unconditional love was given as “Unconditional love is the heartfelt benevolent desire that everyone and everything—ourselves, others, and all that exists in the universe—reaches their greatest possible fulfillment, whatever that may prove to be. This love is freely given, with no consideration of merit, with no strings attached, with no expectation of return, and it is a love that motivates supportive action in the one who loves.” Following this definition, we asked participants to use a five-point scale (from “never” to “a great deal” [0 to 5 points]) with a sixth possibility of “not applicable” (worth 0 points) to respond to four questions: “To what extent do you feel unconditional love toward yourself?,” “…toward other humans?,” “…toward animals?,” “…toward the device on which you are completing this survey?” The total score was the sum of the scores for all questions. In the present study, test-retest validity averaged 0.72.
(4) An overall wellbeing question (“How would you rate your overall physical, emotional, and spiritual wellbeing over the past 3 days?” with a five-point scale: “worst I’ve ever felt,” “pretty bad,” “fair,” “really good,” “best I’ve ever felt.” Scores were 1 (“worst”) to 5 (“best”). This is the first usage of this measure and it is not yet validated relative to other wellbeing measures. In the present study, test-retest validity averaged 0.51.

Calculated scores on these four surveys comprised our dependent variables; note that two of them (physical symptoms of stress scale and the overall wellbeing measure) are not validated with respect to other measures. Thus we refer to results related to these two measures as “measures” or “scores” rather than directly inferring that they represent actual reflections of physical symptoms of stress and overall wellbeing.

#### Qualitative Feedback Survey (Study Day 26)

This survey asked the following questions to elicit feedback on the study and the technology. “write about your experience of this pilot study over the past 26 days” (open text field), “let us know at least one thing you thought was good about how this study was conducted” (open text field), “let us know anything you thought we could have done better” (open text field), “in the future, would you be interested in using technology like the technology you used in this study?” (Yes/No), “if Yes, why?” (open text field), “if No, why not?” (open text field), “What would you do to change the technology so it would be easier to use, more interesting, more engaging, or more positive for you as a user?” (open text field), “What would you suggest we do to reach more people with an improved version of this technology?” (open text field).

### Data Analysis

#### Quantitative Analyses

All quantitative analyses were performed using Libre Office Calc, R 4.1.0, Matlab R2018b, and the mediation bootstrapping program for R with 1000 simulations per model (Tingley et al., 2014). We had no hypothesis about the linearity (or lack thereof) of the changes in the dependent variables over time, so instead of using one-way repeated measures ANOVAs, we compared final (day 25) and initial (day 2) values for each of the dependent variables, at times creating a difference score (first-to-last value; day 25 minus day 2). We compared initial and final values with paired *t*-tests and compared differences between means of separate groups with independent *t*-tests. We did not perform Bonferroni correction of statistical tests on dependent variables used to test the hypotheses, as all of these comparisons were planned. Alpha was set at 0.05 for significance testing, and while all tests were performed on raw (not scaled) data, the scaling factor was linear and would not have changed the results of the statistical tests used. Scaling (dividing each score by the highest possible score) was only used in tables and graphs for ease of comparison between non-calculated dependent variables [i.e., scaling was used for all dependent variables except the deviation from balanced time perspective (dBTP) measure (Jankowski et al., 2020)].

Most analyses were straightforward, but the deviation from balanced time perspective (dBTP) measure was taken from a recent analysis indicating that the time perspective most closely associated with wellbeing is one described by this equation, dubbed by the authors the Deviation from Balanced Time Perspective-revised, though we generally refer to this measure as dBTP:


(1) $$\text{dBTP-r\_noPH}=\sqrt{\left({\left[1-P\,N\right]}^{2}\,{\left[5-P\,P\right]}^{2}\,{\left[1-P\,F\right]}^{2}\,{\left[5-F\right]}^{2}\right)}$$

PN and PP indicate each participant’s past negative and past positive scores (respectively), PF and F indicate each participant’s present fatalistic and future scores (respectively). The numbers 1 and 5 indicate the most adaptive (“balanced”) score for each time perspective factor (example: it is most adaptive for overall wellbeing to think very few negative thoughts about the past [PN = 1] and many positive thoughts about the past [PP = 5]). Note that DBTP-r_noPH = 8 for a participant with the least adaptive time perspective (all values as the four factors are at the extreme end of the spectrum relative to the desired values), and will be zero for a participant with a “perfectly balanced” time perspective. We choose to remove present hedonic (PH) values as a factor, due to their non-linear and apparently inconsistent relationship with overall wellbeing (Jankowski et al., 2020). We indicate this in equation 1 by adding “_noPH” to the signifier at the left of the equation.

#### Qualitative Analyses

We used a thematic analysis approach. Coding and theme extraction was performed by two experienced researchers skilled in assessing qualitative data, but of course bias will be present in terms of which ideas, feedback, and experiences are reported as examples, and how codes and themes are selected. To mitigate the impact of this bias, where possible, any quantitative results obtained from the qualitative feedback survey are also provided in table form. Further, all raw data are available on request.

## Results

### COVID Pivot

Our original plan was to facilitate 10 in-person focus groups across the United States and Canada throughout 2020, integrate feedback from those groups into iterative designs, and create a blueprint for developers to write software. To find our design partners, we reached out to 26 clinicians working with populations at the intersection of addiction, abuse, incarceration, poverty and trauma across the United States and Canada, including leaders in the Latino Social Workers Organization and the National Association of Black Social Workers. Our intention was to schedule in-person paid focus groups with their populations; 13 of these mental health professionals showed sustained interest.

However, soon travel and physical proximity restrictions imposed by COVID-19 resulted in a need to pivot our plans. We moved most of our participant recruitment online to an online work provider allowing pre-screening for desired demographics. We streamlined our approach and conducted a beta-test online focus group with unpaid beta testers and seven formal online focus groups. These focus groups introduced participants to the study and the motivations behind it. As a result of this change we were able to reach vulnerable populations with a potentially useful intervention and also provide extra income (up to $180 for full participation, see “Materials and Methods”). We refer to the participants who completed the 26-day study as *design partners*, because their feedback helped us iterate Time Machine’s design.

### Design Partner Demographics

By March 3, 2021, of 104 design partners enrolled, 97 design partners completed the study and were paid the full amount (93% completion rate). Of these, only 6 design partners took longer than 30 days to complete the study, with the longest time frame being 36 days. The remaining design partners did not ask for partial payment or communicate with us to say they dropped out, even after we contacted them to remind them they could receive partial payment. Our analyses included 96 of these design partners because values from one design partner did not register in the database due to a technical error. Demographics for the 96 included design partners are shown in Table 1. We did not reach our goal of at least 50% BIPOC design partners; in fact, 74% of our design partners were white. We did reach our goal of finding a group of design partners with life experiences related to LGBTQ+ identification (24%), disabilities (26%), incarceration (8.3%), military service (4.2%), and adverse childhood events (64%).

**TABLE 1:** Demographics for the 96 design partners in the study.

Self-reported race/ethnicity	Total	Women	LGBTQ+	Disabilities	Incarceration	Served in United States military	ACES score 2+
Asian	3	1	1	0	0	0	3
Native American/White	2	2	0	1	0	0	2
Black	9	4	2	0	0	0	5
Black/LatinX	3	0	0	1	0	0	1
LatinX	4	3	1	2	0	1	2
Mixed	2	0	0	1	1	0	2
South Asian	2	0	0	1	0	0	2
White	71	50	19	19	7	3	44
*Total*	96	*60*	*23*	*25*	*8*	*4*	*61*

*Note that the questions on the demographic survey that asked about race/ethnicity and gender provided an open text field for responses, so individuals could self-identify. Zero individuals self-described as non-binary or transgender, but it is possible that some of those who self-identified as women or men are also other genders.*

### Unpaid (Optional) Engagements

During the 10 days following the required task days, 86 of our 96 design partners (90%) chose to engage with Time Machine during this unpaid optional period of the study. For those who chose to engage with the technology, the mean number of days of engagement was 5.8 days. Two early participants engaged in the recording task during the optional period for longer than 10 days (11 and 14 days) before we realized there was a bug in the software that did not let them know the optional period was over; we included their data in all analyses regardless of this deviation from the study plan.

### Experimental Wellness Sliders

**FIGURE 3:**
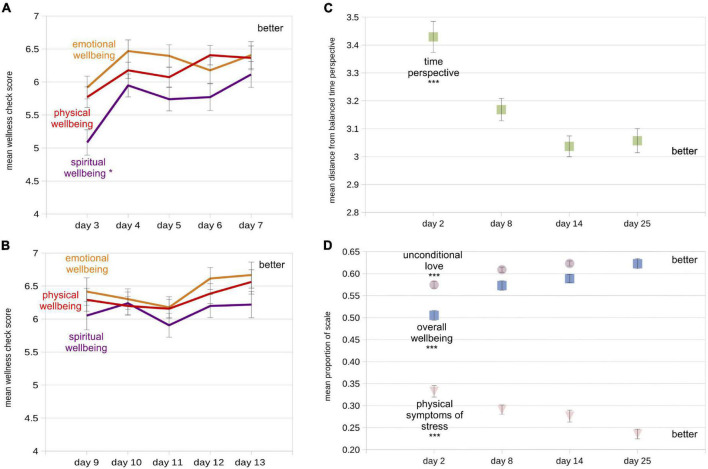
Mean values of wellbeing measures at all measurement times (*N* = 96). “Better” label indicates the area of the plots indicating improvement (higher or lower values, depending on measure). **(A)** Mean responses from experimental within-app wellness checks (1 = worst, 10 = best) for the first five task days. **(B)** Same as **(A)**, but for the second five task days. **(C)** Mean distance from balanced time perspective (dBTP) on each of the four recurring assessments. **(D)** Mean proportion of the total scale used for the unconditional love (violet circles), overall wellbeing (blue squares), and physical symptoms of stress (pink triangle) measures. Lighter colors are used so small error bars inside the symbols can be seen. Also note that the day 25 value for unconditional love is the same as the overall wellbeing value (also see Table 2), so one symbol occludes the other. Error bars in each figure represent +/- 1 standard error of the mean (S.E.M), calculated *within* participants. For first-vs.-last measure comparison statistics (paired *t*-tests), *p < 0.05; ***p < 0.0001.

We examined responses to the experimental wellness sliders (Figure 2) registered within the app during the two 5-day required task periods and their relationship to other study variables. First, we found increases in measures of physical and spiritual wellness over time; significant self-reported increases occurred only during the first 5-day period (Figures 3A,B; 1st 5 days: physical: *t*_95_ = 2.07, *p* < 0.045, *d* = 0.21; emotional: *t*_95_ = 1.60, *p* < 0.113, *d* = 0.16; spiritual: *t*_95_ = 3.38, *p* < 0.002, *d* = 0.35; 2nd 5 days: all *p*-values > 0.30). As these were not our planned analyses, we performed Bonferroni correction on these results, after which only the data from the spiritual wellness slider can be considered significant (cutoff = *p* < 0.008). Thus it appears that measures of whatever design partners defined as spiritual health increased over the course of the first 5 required task days. Note that the mean test-retest reliability of the spiritual wellbeing slider was moderate-to-good: 0.48 over the first five days, and 0.41 over the second five days.

Because we were using these sliders to solicit responses for the first time, we were curious whether the data reported using these sliders would correlate with data from the dependent variables assessed with the recurring assessments, as a way of validating the sliders. To perform these correlations, we first considered which day or days would be appropriate to compare – especially given that questions on the recurring assessments asked design partners to consider the most recent 3 days (including the survey day) in their responses, while the sliders asked only about the current day. Because of this consideration, we determined that the average scores on the last 2 days of slider responses would be most appropriate to compare to the responses on the recurring assessments. Thus we used Pearson correlations to compare the average of each slider’s responses for the 2 days prior to recurring assessment 2 (given on study day 8) and recurring assessment 3 (given on study day 14) with the four dependent variables calculated from recurring assessment responses on study days 8 and 14.

Overall, many of these correlations were significant, and a clear pattern emerged indicating that design partners were, for the most part, giving the three sliders equivalent meanings (Table 2). Averages of the two slider responses prior to the recurring assessments were significantly negatively correlated with dBTP and physical symptoms of stress scores on the recurring assessments. Correlations between slider responses and overall wellbeing were strong and positive, with a slight shift toward a higher correlation with emotional wellbeing for data from the third repeated assessment. Interestingly, the odd dependent variable out was the unconditional love variable – there were no significant correlations between average responses on any of the three sliders and repeated assessment measures of unconditional love. The lack of correlations between reported feelings of unconditional love and responses on the three sliders suggests that design partners did not feel that the unconditional love measure had any consistent relationship to physical, emotional, or spiritual wellness; thus the feelings of unconditional love questions on the repeated assessments seem to address a unique variable. Further, it appears that responses on the three sliders were for the most part consistently tied to time perspective, physical symptoms of stress, and overall wellbeing. These results also indicate that design partners were relatively intentional in their responses both within (sliders) and outside of (recurring assessments) the Time Machine technology.

**TABLE 2:** *R* values for Pearson correlations between slider responses (average of the 2 days of responses prior to repeated assessments) vs. dependent variables on repeated assessments 2 and 3.

Measure	vs. physical	vs. emotional	vs. spiritual
dBTP day 8/RA2 day 14/RA3	−**0.255** −**0.308**	−**0.301** −**0.371**	−**0.210** −**0.337**
PSS day 8/RA2 day 14/RA3	−**0.392** −**0.351**	−**0.351** −**0.233**	−**0.327** −**0.260**
UL day 8/RA2 day 14/RA3	0.139 0.110	0.129 0.176	0.157 0.135
Overall day 8/RA2 day 14/RA3	**0.617** **0.517**	**0.596** **0.575**	**0.561** **0.534**

*RA, repeated assessment; dBTP, distance from balanced time perspective; PSS, physical symptoms of stress; UL, feelings of unconditional love; overall, overall wellbeing. Bolded values are significant.*

### Pre-planned Hypothesis Tests on Dependent Variables

All four dependent variables evidenced significant improvement from the initial (day 2) to final (day 25) values (Table 3A and Figures 3C,D), supporting Hypothesis 1 (Methods). The most impressive results were a 12% improvement in the measure of overall wellbeing from the first to last assessment, and a near 10% drop in the measure of physical symptoms of stress during the same time period. For all four dependent variables, the most quantitative improvement in mean values occurred between the first and second assessments, although for the physical symptoms of stress measure, the improvements at the beginning and end of the study were roughly equivalent (improvement of 0.385 from the first to second assessment, and 0.376 from the third to last assessment), hinting at a potentially different mechanism underlying changes in scores on this measure.

**TABLE 3:** Mean (SD) values for each of the raw dependent variables on all four assessments as well as statistics for paired *t*-tests comparing values on first and last assessments within participants.

**Measure**	**Day 2 RA 1**	**Day 8 RA 2**	**Day 14 RA 3**	**Day 25 RA 4**	**First-to-last change RA** **4 – RA 1**	***t***	***p***
**(A) Data from 96 participants taken together.**							
dBTP	3.43 (1.04)	3.17 (1.02)	3.04 (0.99)	3.06 (1.10)	−0.37 (0.85) prop: −0.05 (0.11)	*t*_95_ = 4.29	**<0.0001**
PSS	0.33 (0.26)	0.29 (0.24)	0.28 (0.24)	0.24 (0.24)	−0.10 (0.20)	*t*_95_ = 4.77	**<0.0001**
UL	0.57 (0.14)	0.61 (0.15)	0.62 (0.16)	0.62 (0.16)	0.05 (0.12)	*t*_95_ = 4.01	**<0.0002**
Overall	0.51 (0.18)	0.57 (0.17)	0.59 (0.15)	0.62 (0.17)	0.12 (0.18)	*t*_95_ = 6.21	**<0.0001**
**(B) Data split according to group (task order).**							
dBTP							
Recording first	3.48 (1.01)	3.10 (1.12)	2.89 (1.04)	2.91 (1.13)	−0.56 (0.85) prop: −0.07 (0.11)	**t*_51_* = 4.78	**<0.0001**
Quote first	3.37 (1.08)	3.25 (0.89)	3.21 (0.90)	3.22 (1.04)	−0.15 (0.80) prop: −0.02 (0.10)	*N.S.*	>0.230
PSS							
Recording first	0.29 (0.24)	0.24 (0.22)	0.23 (0.20)	0.20 (0.20)	−0.09 (0.21)	**t*_51_* = 3.15	**<0.003**
Quote first	0.38 (0.27)	0.35 (0.25)	0.33 (0.28)	0.28 (0.27)	−0.10 (0.19)	**t*_43_* = 3.63	**<0.0008**
UL							
Recording first	0.57 (0.11)	0.62 (0.14)	0.63 (0.15)	0.63 (0.16)	0.06 (0.13)	**t*_51_* = 3.55	**<0.0009**
Quote first	0.58 (0.17)	0.60 (0.16)	0.61 (0.17)	0.61 (0.16)	0.03 (0.10)	**t*_43_* = 1.96	<0.058
Overall							
Recording first	0.52 (0.17)	0.61 (0.15)	0.60 (0.15)	0.65 (0.17)	0.13 (0.18)	**t*_51_* = 5.34	**<0.0001**
Quote first	0.49 (0.19)	0.53 (0.19)	0.57 (0.15)	0.59 (0.18)	0.10 (0.19)	**t*_43_* = 3.40	**<0.002**
**(C) Data split according to median split on adverse childhood experiences survey (ACES scores).**							
dBTP							
ACES < 3	3.18 (1.05)	2.95 (1.08)	2.86 (0.99)	2.79 (1.17)	−0.39 (0.93) prop: −0.05 (0.12)	**t*_51_* = 3.05	**<0.004**
ACES ≥ 3	3.71 (0.96)	3.42 (0.89)	3.24 (0.96)	3.36 (0.93)	−0.35 (0.76) prop: −0.03 (0.10)	**t*_43_* = 3.04	**<0.004**
PSS							
ACES < 3	0.33 (0.24)	0.28 (0.24)	0.25 (0.26)	0.22 (0.23)	−0.11 (0.20)	**t*_51_* = 4.14	**<0.0002**
ACES ≥ 3	0.33 (0.28)	0.30 (0.25)	0.30 (0.22)	0.25 (0.26)	−0.08 (0.20)	**t*_43_* = 2.60	**<0.02**
UL							
ACES < 3	0.58 (0.12)	0.62 (0.13)	0.62 (0.14)	0.64 (0.15)	0.05 (0.12)	**t*_51_* = 3.13	**<0.004**
ACES ≥ 3	0.57 (0.16)	0.60 (0.16)	0.62 (0.17)	0.61 (0.17)	0.04 (0.11)	**t*_43_* = 2.50	**<0.02**
Overall							
ACES < 3	0.54 (0.16)	0.58 (0.18)	0.61 (0.14)	0.62 (0.19)	0.08 (0.19)	**t*_51_* = 2.97	**<0.005**
ACES ≥ 3	0.46 (0.18)	0.57 (0.17)	0.57 (0.15)	0.62 (0.16)	0.16 (0.17)	**t*_43_* = 6.20	**<0.0001**

*RA, repeated assessment; first-to-last change = mean difference of RA4 minus RA1 values; dBTP, distance from balanced time perspective; prop., proportion of total scale as comparison measure for dBTP, all other values given in proportion of total scale; PSS, physical symptoms of stress; UL, feelings of unconditional love; overall, overall wellbeing. Bold *p*-values indicate significance.*

**FIGURE 4:**
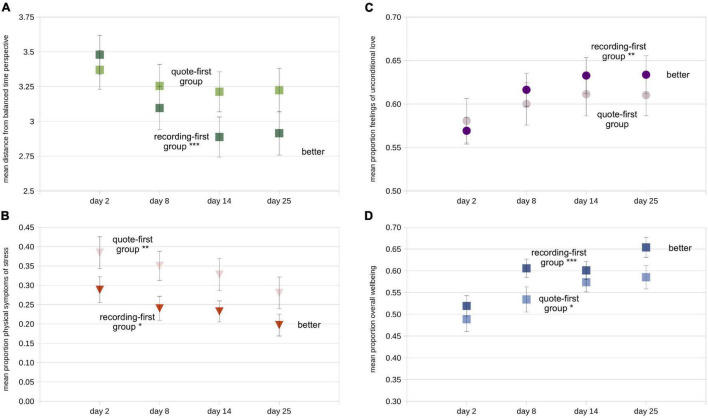
Mean values of all four dependent variables split according to task order (darker symbols = recording-first group; lighter symbols = quote-first group). **(A)** Mean distance from balanced time perspective (dBTP) on each of the four recurring assessments. **(B)** Mean proportion of the total scale used for the physical symptoms of stress measure, **(C)** the unconditional love measure, and **(D)** the overall wellbeing measure. Error bars in each figure represent +/- 1 S.E.M, calculated across participants. For first-vs.-last measure comparison statistics (paired *t*-tests), *p < 0.05; **p < 0.01; ***p < 0.0001.

Examining the same data split according to task order (group) provides evidence that the order of the tasks had some influence on each of the four dependent variables, generally favoring the recording-first group in partial support of Hypothesis 2 (Figures 4A–D and Table 3B). Note that we did not combine data across groups for each of the required tasks because of the clear indication, discussed above, that the first 5 days showed the most improvement in most measures regardless of the nature of the task. The improvements in time perspective from the first to last assessment were significantly larger in the recording-first versus the quote-first group (day 25 minus day 2: *t*_94_ = 2.46, *p* < 0.016, *d* = 0.51), as predicted. Overall, the recording-first group significantly improved on the dBTP first-to-last measure from the first to the last assessment, while the quote-first group did not (day 2 to day 25: recording-first group *t*_51_ = 4.78, *p* < 0.0001, *d* = 0.66; quote-first group *p* > 0.231; Figure 4A). Meanwhile, the physical symptoms of stress measure was quantitatively lower for the recording-first group throughout the study. Because on day 2 participants were told which group they were to be placed in prior to taking the survey, it is possible that this influenced some participants in the quote-first group to be particularly aware of their physical symptoms of stress in response to not being able to perform the time-travel process first. While there was no significant difference in improvement on the physical symptoms of stress measure between the groups (*p* > 0.75), both groups improved significantly, showing reductions in physical symptoms of stress from first-to-last assessment (recording-first group *t*_51_ = 3.15, *p* < 0.003, *d* = 0.44; quote-first group *t*_43_ = 3.63, *p* < 0.0008, *d* = 0.55; Figure 4B). Improvements in feelings of unconditional love from pre-to-post assessment were not significantly different between the groups either (*p* > 0.15). Nevertheless, the recording-first group showed quantitatively greater improvement while improvement in the quote-first group was only borderline significant (recording-first improvement = 1.29, *t*_51_ = 3.67, *p* < 0.0006, *d* = 0.49; quote-first improvement = 0.59, *t*_43_ = 1.96, *p* < 0.057, *d* = 0.29; Figure 4C). Finally, the overall wellbeing measure was also not significantly different between groups (*p* > 0.31). Both groups improved significantly on the overall wellbeing measure between the first and last assessments (recording-first group *t*_51_ = 5.34, *p* < 0.0001, *d* = 0.71; quote-first group *t*_43_ = 3.40, *p* < 0.002, *d* = 0.51; Figure 4D). Critically, participants were not assigned by the experimenters to a particular task order, but were instead randomly assigned by the software and there were no significant differences between the groups on any dependent variable on the initial assessment or ACES scores (all *p*s > 0.070). Overall, these results suggest that recording-first participants had a slight advantage in that their improvements were more impressive than the improvements of quote-first participants. Particularly for dBTP and feelings of unconditional love significant improvements from the first to last assessments were absent or borderline for the quote-first group, while they were robust for the recording-first group. However, these results can only be seen as partial support for Hypothesis 2, which asserted that *all* dependent variables would show more improvement amongst participants in the recording-first group.

**FIGURE 5:**
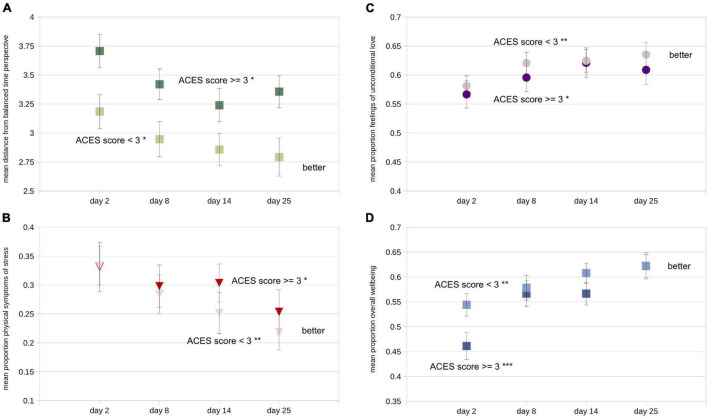
Mean values of all four dependent variables split according to median split on ACES inventory score (darker symbols = ACES score ≥ 3; lighter symbols = ACES score < 3). **(A)** Mean distance from balanced time perspective (dBTP) on each of the four recurring assessments. **(B)** Mean proportion of the total scale used for the physical symptoms of stress measure, **(C)** the unconditional love measure, and **(D)** the overall wellbeing measure. Error bars in each figure represent +/- 1 S.E.M. calculated across participants. For first-vs.-last measure comparison statistics (paired *t*-tests), *p < 0.05; **p < 0.01; ***p < 0.0001.

Because all of our data were based on some type of self-report, we were especially interested in whether the number of reported adverse childhood experiences on the ACES survey would be reflected in the dependent variables, at least on the first assessment day, as predicted by Hypothesis 3. Because of the strong literature in this area (see Introduction), if there were no differences in dependent variables related to ACES scores, we would suspect either that participants were not being careful and honestly reporting their experiences on the ACES survey, the repeated assessments, or both. While a median split (low ACES < 3; high ACES ≥ 3) indicated that on all four measures low and high ACES design partners showed significant improvements from the first to the last recurring assessment on all four measures (Table 3C), there were clear ACES-related differences for both the dBTP and overall wellbeing measures. On the dBTP measure, design partners reporting higher ACES (dark symbols in Figure 5A) had less balanced time perspectives (less adaptive) compared to design partners reporting lower ACES (lighter symbols) on all 4 days (day 2: *t*_94_ = 2.53, *p* < 0.015, *d* = 0.52; day 8: *t*_94_ = 2.33, *p* < 0.025, *d* = 0.48; day 14: *t*_94_ = 1.92, *p* < 0.060, *d* = 0.39; day 25: *t*_94_ = 2.59, *p* < 0.015, *d* = 0.53); the day 2 results support Hypothesis 3. In contrast, neither the scores on the physical symptoms of stress measure nor the unconditional love measure revealed differences between higher and lower ACES individuals, though the general tendency was for lower-ACES individuals to give more psychologically positive responses on both measures than higher ACES individuals (Figures 5B,C). Overall wellbeing scores were significantly higher on the first recurring assessment day for those with relatively lower ACES scores as compared to those with higher ACES scores (Figure 5D; day 2: *t*_94_ = 2.34, *p* < 0.025, *d* = 0.48), in support of Hypothesis 3. In our most striking quantitative result, this difference disappeared during the study period, with both groups sharing very similar mean scores on the overall wellbeing measure on the day of the final recurring assessment, such that design partners with lower ACES improved significantly less (half as much on average) on their overall wellbeing scores than design partners with higher ACES (*t*_94_ = 2.23, *p* < 0.028, *d* = 0.46). While both groups showed significant improvement on the overall wellbeing measure (higher ACES: *t*_44_ = 6.36, *p* < 0.001, *d* = 0.95; lower ACES: *t*_50_ = 2.94, *p* < 0.005, *d* = 0.41), those with higher ACES reported a 16% improvement on the overall wellbeing measure over the course of the study, the largest change we recorded. There were no significant differences in improvement for any other dependent variable. Thus the results from the time perspective and overall wellbeing measures support Hypothesis 3, while the results from the overall wellbeing measure provide strong evidence against Hypothesis 4 – which was that individuals with lower ACES scores would report *greater* improvement on all four dependent variables. In addition to addressing our hypotheses, the varying patterns of these results support two additional ideas: (1) our design partners were for the most part accurately recording their experiences, and (2) something about the study improved all four dependent variables for individuals with both lower and higher ACES scores, with striking improvement on the overall wellbeing measure for those with lower ACES scores.

To investigate Hypothesis 5, which predicted that greater use of the technology during the study period would positively relate to greater improvement in all dependent variables, we examined the relationships between three independent variables and changes in our dependent variables. To this end, we performed linear regressions on differences calculated between the first and last repeated assessment day for each dependent variable as predicted by all three independent variables (ACES score, group, number of optional logins). These four multiple linear regressions yielded significant results for the dBTP and overall wellbeing measures only (Table 4). In the reduced models, improvement in dBTP (i.e., a reduction in value) was positively predicted by inclusion in the recording-first group and a greater number of optional logins, while the improvement in overall wellbeing was positively predicted by those same independent variables as well as a higher ACES score. The number of optional logins was included in both reduced models, providing additional evidence that the recording and wellness check tasks available in the optional period of the study influenced both time perspective and wellbeing scores positively. Thus these results partially support Hypothesis 5, at least for improvements in time perspective and the overall wellbeing measure.

**TABLE 4:** Results from multiple linear regressions predicting changes (RA4 value minus RA1 value) in the four dependent variables.

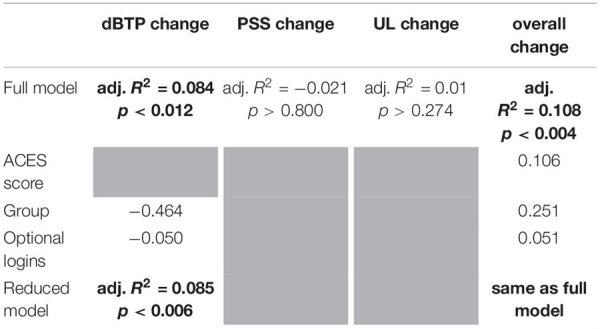

*Predictors are independent variables (across top). RA, repeated assessment; dBTP, distance from balanced time perspective; PSS, physical symptoms of stress; UL, feelings of unconditional love; overall, overall wellbeing. Bold adjusted *R*^2^ values indicate significance. Estimates for each participating factor in the reduced model are shown, bolded estimates indicate independent significance for the given factor. Rows marked “reduced model” give results for the reduced model as a whole, where the full model was significant. Shaded cells indicate the factor was not included in the reduced model because the adjusted *R*^2^ improved when the given factor was removed, or that a reduced model was not created due to insignificance of the full model. Note that group was coded as 0 (for quote-first group) and 1 (for recording-first group), so a positive estimate indicates that higher values of the dependent variable were correlated with the recording-first group. Also note that for dBTP and PSS, negative changes between RA1 and RA4 indicated improvement, while the reverse is true for UL and overall change measures.*

### *Post-hoc* Analyses of Dependent Variables

The pre-planned tests of our five hypotheses revealed a pattern of results that warranted further examination. Because improvements in time perspective and scores on the overall wellbeing measure were the only dependent variables that were related to the use of the technology during the non-required period, we first focused on determining whether changes in the other dependent variables occurring during the required portion of the study would predict first-to-last changes in dBTP and the overall wellbeing measure. Thus we used as predictors the difference between scores on days 14 and 2 (i.e., day 14 minus day 2) for the physical symptoms of stress and unconditional love measures. Full models for each were significant (dBTP: adj. *R*^2^ = 0.051, *p* < 0.04; overall wellbeing: adj. *R*^2^ = 0.058, *p* < 0.03). Reduced models only included a single factor – changes in feelings of unconditional love. In both cases increases in unconditional love were predictive of improvement in both dBTP (i.e., reduction in dBTP; adj. *R*^2^ = 0.060, *p* < 0.01) and the overall wellbeing measure (i.e., increase in overall wellbeing scores; adj. *R*^2^ = 0.063, *p* < 0.008). If the relationships between changes in feelings of unconditional love and both dependent variables were moderated by the number of optional logins between the third and fourth repeated assessments, this would be a further indication that the tasks performed during the optional logins (recording/listening to messages and performing wellness checks) were related to improvements in time perspective and overall wellbeing scores. In fact, moderation analyses indicated that increases in overall wellbeing scores were significantly positively moderated by the number of optional logins, while decreases in dBTP values were not moderated by the same (model included unconditional love score day 3 minus day 1 [UL3-1], optional logins, and their interaction: adj. *R*^2^ = 0.098, *p* < 0.004 for overall wellbeing change; adj. *R*^2^ = 0.075, *p* < 0.01 for dBPT change; moderation by number of optional logins: *p* < 0.04 for overall wellbeing change; *p* > 0.10 for dBPT change). These results suggest that changes in unconditional love in the required portion of the study predict study-long changes in both dBTP and overall wellbeing scores, and for overall wellbeing this relationship was stronger for individuals who logged in more often during the optional period. While this result lends validity to both the overall wellbeing measure as well as the value of the recording task performed during the optional period, it is worth noting that there is no implied causality with any moderation effect.

**FIGURE 6:**
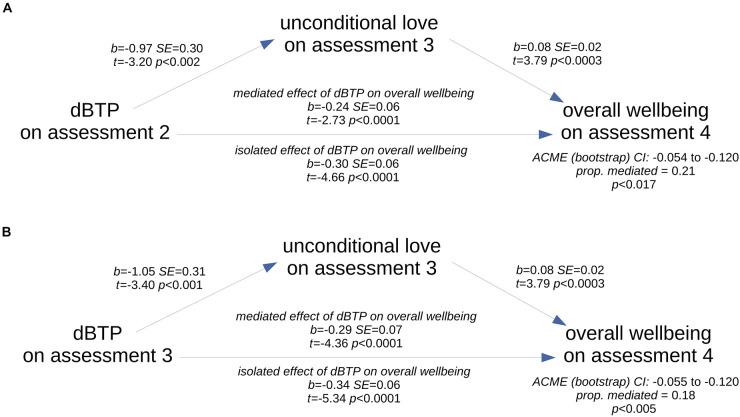
Schematics of the two significant mediation relationships. Scores on the unconditional love measure on the third assessment partially mediated the relationship between scores on the dBTP measure on the second **(A)** and third **(B)** assessments and overall wellbeing scores as measured on the last assessment.

To examine potentially causal relationships between the three apparently related dependent variables – feelings of unconditional love, dBTP and overall wellbeing scores – we performed four mediation analyses. For each, overall wellbeing on the fourth assessment (day 25) was the dependent variable, the unconditional love score on the third repeated assessment (day 14) was the mediator, and the treatment was either the physical symptom of stress (PSS) score or the dBTP score calculated from the third or second repeated assessments (both the dBTP and PSS factors from a given assessment time were included in the model, but only one was the treatment in each of the mediation analyses). Scores on given days were selected instead of change scores because for mediation analysis to suggest a causal relationship, the treatment and the mediator should be measured prior to the outcome variable, and change scores required measurement on the first assessment day (day 2). Unconditional love was selected as the mediating variable because it showed a clear relationship with overall wellbeing scores (see above), and prior research indicated that self-transcendent emotions can mediate wellbeing (Vieten et al., 2014). Starting with scores on the third assessment (following the required portion of the study), there was no mediation by the unconditional love measure for the model with physical symptoms of stress measure as the treatment (average causal mediation effect [ACME] *p* > 0.929 for PSS on third assessment), but for the model with the dBTP measure as the treatment, there was significant partial mediation by the unconditional love measure (ACME 95% CI −0.055, −0.120; *p* < 0.005, est. prop. mediated 0.182). Note that the negative value for the ACME arose because lower values of dBTP (more balanced time perspective) on the third assessment (day 14) positively predicted both greater feelings of unconditional love on the third assessment as well as greater overall wellbeing scores on the final assessment (day 25), while the unconditional love measure positively predicted scores on the wellbeing measure (Figure 6A). Further, the same was true for dBTP on the second recurring assessment (Figure 6B; ACME 95% CI −0.054, −0.120; *p* < 0.017, est. prop. mediated 0.207), but note that this result does not pass Bonferroni correction (Bonferroni cutoff = *p* < 0.0125). When physical symptoms of stress on the second assessment was the treatment, no mediation was present (ACME *p* > 0.963).

Finally, to determine whether the unconditional love mediation effect was driven by changes related to the study rather than any pre-existing relationship between time perspective and overall wellbeing, we examined this same mediation using as the treatment factor the dBTP measure on the first recurring assessment. We found the mediation was not present (ACME *p* > 0.311), even though dBTP on the first assessment strongly predicted the final overall wellbeing score (adj. *R*^2^ = 0.078, *p* < 0.004). Thus these results support the idea that the mediation effect was driven by changes in the relationship between dBTP, unconditional love, and overall wellbeing scores that occurred during the time of the study. Overall, these mediation analyses results point to a causal relationship in which unconditional love feelings apparently influenced the relationship between time perspective and overall wellbeing scores.

### Qualitative Findings

#### Inclusive Design Process

In our unique study design, we held the study and technology features described above (Methods) constant while we incorporated aesthetic enhancements requested by our design partners. We based these enhancements on early feedback from attendees at an unpaid focus group as well as feedback from paid focus group participants who met within the first month of the study (September 2020). As a result of this early feedback, we altered the software to include: a recording playback button (due to background noise and privacy issues, many design partners were whispering and wanted to make sure their recordings were audible; added after 34 participants had started), the ability to cancel recordings (in case they wanted to re-think their message; added after 34 participants had started), a countdown timer for recordings (so they could time their recording to the 1-min limit; added after 34 participants had started), better daily notifications (some were not being received; added after 46 participants had started), a study-day tracker to remind people where they were in the study (so they knew when each study stage would start/end; added after 46 participants had started), and a table showing previous “wellness check” responses (because design partners wanted to track their changes in wellness over time; added after 46 participants had started). While none of these enhancements impacted whether design partners could perform the study tasks, we do think they enhanced the technology and also reminded our design partners of their role as key team members in improving the technology. The impact of this engagement is not to be underestimated as a potential factor in the overall results (see section “Discussion”).

This level of engagement seemed to be contagious. For example, one Turker, known for organizing other Turkers, went out of their way to recruit others for this study because they were so impressed by their own experience in it. Further, three sets of Turkers requested unscheduled and impromptu focus groups following the close of their paid participation. When they were told they would not be paid for these groups and we could not report their feedback from these groups in any scholarly paper, the Turkers still wished to connect via Zoom. Some of them chose to show their faces and share their voices. They offered helpful feedback that we are using to shape the next stage of the technology. However, the most exciting outcome of those impromptu focus groups is that the design partners who organized them asked if they could continue to use the technology after the study was complete. We realized we could offer this to all design partners, and added an option at the end of the study; eight design partners continued using the technology following the end of the study.

#### Feedback Survey Results

The final survey comprised eight questions with open-ended text-field responses and one yes/no question. As one measure of engagement with the study, we calculated the average time taken to complete the final survey. After removing two obvious outliers for whom it was clear browsers were left open as they took the survey, the average time taken was 16.92 min. Since the survey could be completed with concise answers in less than 4 min and payment did not depend on survey answers, this indicated to us that on average, our design partners were invested in communicating their actual experiences. Further, their responses to key questions indicated this investment as well. For instance, the yes/no question, “In the future, would you be interested in using an improved version of the technology you used in this study?” was answered in the negative by only 4 of the 96 design partners. Reasons for these negative responses were given by all four, in response to the follow-up question, “briefly describe why you wouldn’t be interested in using technology like this in the future.”

• “Because I really rather focus on God than myself. I do feel that being in touch with all parts of myself is helpful, but it’s not really something to focus on for too long. God lives outside of time and I can connect to Him the same, whether it’s my past, present of future self. Also, I don’t like talking out loud or listening to myself because I’m rarely alone in the house.”
• “Because I don’t feel that it helped me in any significant way.”
• “Because I don’t want to check in with my wellbeing by looking at a phone or computer screen.”
• “Because I don’t think it’s powerful enough to deal with the issues I have in my life.”

Representative responses from the remaining 92 design partners when asked, “briefly describe why you would be interested in using technology like this in the future,” include the following:

• “I just think it was super helpful and if it can help someone else, even better.”
• “Because I often overlook my own mental health, and need to create better habits to support my general wellness. I am used to putting my needs last, and focusing on the needs of others first.”
• “I’d like to see how we shaped it together after all the feedback we’ve shared, plus I really enjoy using it.”
• “Because I want to see if you took my suggestions :-)”
• “Because I got used to looking forward to take one minute a day to actually think seriously about how I’m feeling.”
• “Some days I really do need to remind myself of things I’ve already been through, or how things will be better in the future. It’s hard to see through the trees sometimes without a little push.”

To perform the thematic analysis without concern for how each question might bias responses, we coded each statement given in response to any of the questions according to two codes: positive and negative. Because the qualitative feedback survey asked an equal number of questions requiring positive and negative responses, the survey itself should not have biased our design partners to produce more positive or negative responses. Once coding was done, we extracted three overarching themes in the comments: the technology, the study design, and overall response to the study. Table 5 reports the total number of statements within each theme, as well as within sub-themes.

**TABLE 5:** Results of qualitative analysis of the statements on the feedback survey.

	Negative experiences	*N*	Positive experiences	*N*
**Technology**				
	didn’t like the visuals	7	appreciated reminders/notifications	3
	wanted more freedom in recording time, availability of app	12	liked flexibility in timing	4
	needed prompts/more guidance for recording	17	liked brief recording time limits	4
	wished for better reminders/notifications	25	felt technology was interesting/unique	20
	had periodic tech issues (operating system, sound, other)	28	tech seemed easy/quick to use	38
	**Total technology – negative**	**89**	**Total technology – positive**	**69**
**Study design**				
	wanted more meditations	3	enjoyed guided meditation in focus group	10
	felt the need for greater control over which task was performed	5	seemed like study taught ideas/tools to use going forward	10
	wanted greater variety within wellness checks	6	felt positive about study team	31
	would have appreciated more group interactions	11		
	was hoping for more guidance in the study overall	17		
	wanted a greater variety of tasks	19		
	**Total study design – negative**	**61**	**Total study design – positive**	**51**
**Response to study itself**				
	didn’t like talking/listening to self	7	felt calmer	3
	felt the process was not helpful for self/not needed for self	13	increased connection to others	3
			felt similar to journaling	4
			liked visuals/graphics	5
			felt generally positive	5
			gave perspective	7
			grew from not enjoying to enjoying talking to self	7
			originally skeptical of process but changed mind	7
			felt kinder to self	9
			liked listening to self	9
			felt like this process is a good/important idea	11
			liked listening to quotes	11
			looked forward to future	12
			helped mood/mental health	13
			connected with/learned about self	14
			increased loving feelings toward self	18
			generally enjoyed process	34
			seemed generally helpful	35
			felt like a positive daily routine	39
	**Total response – negative**	**20**	**Total response – positive**	**246**

*To read examples of each of the two codes (positive and negative) within each theme (technology, study design, and response to the study), see “Results.”*

Within the technology theme, statements coded as negative included, “The system seemed to miscount the day I was on,” and, “Everything went great, but toward the end, Days 15–24, the Day got stuck at 15 and never went past that, which made it confusing to keep track of what day I was on in the study.” These statements referred to a new feature to report on screen the day participants were on in terms of study progress; the actual database correctly counted the participants’ study days. Positive technology statements included, “I’m a UX designer, and I actually found the technology to be very straight forward and easy to use (but I also recognize that I’m technically savvy…)” and, “it was well spaced out in the fact that you could do it at your pace instead of being rushed to finish it like many other things like this are.”

Within the study design theme, statements coded as negative included, “I think the recordings will be better and maybe easier if you have something think about and spell out which scenario they’re planning to record, and write it down,” and, “The guided meditation was very impactful to me and I think having a bit more direction could have helped - not necessarily something as intensive as the guided meditation, but maybe some prompts for the recordings or reminders of how to approach our past/future selves.” Positively coded study design comments included, “I really liked how responsive and available the team was to hear our issues along the way, and sometimes just to check in and see how we were doing with the project,” and “Inspiring onboarding session with leaders of the study.”

Finally, within the response to the study theme, statements coded as negative included, “I didn’t feel overly compelled to continue using it after the 15 days or so, but I could see how others might benefit from its continued use,” and, “I’ve never been much of a believer in this sort of thing. Sadly, I must report that this experience has not changed that.” Positive study response statements were: “My experience with this study was nothing but positive, starting from the focus group on. The requesters of the study were very responsive, kind and helpful. I have learned a life-long, life-changing skill because of this study and I am very grateful and blessed to have been part of it,” and, “Oh my goodness. I have struggled for so many years with a lot of mistakes and a lot of bad choices. I haven’t been able to think about myself without a bit of hatred for who i used to be. Y’know what? I don’t have those feelings right now. I understand myself now I think. I am able to feel peace now and empathy, which is something I struggle with. Am I where I want to be yet with how I feel about myself? No. But am I on the right track? Yep. I am looking forward to meeting my future self one day I think she is ready for us now too.”

We believe the overwhelmingly positive responses to the study itself are related both to the features of the technology and the relationships we developed with our design partners over the course of the study. Future versions of Time Machine to be released publicly will take into account many of the suggestions we received on the feedback survey (see Discussion).

## Discussion

### Conclusions and Limitations

The 96 paid design partners who completed this 26-day pilot study were largely positive about the technology and the study, as evidenced by 90% participation in the unpaid optional period of the study, the detailed and mostly positive feedback received on the final survey, and the fact that 96% of our design partners said that they would like to use an improved version of this technology again.

Our hypotheses were confirmed (Hypothesis 1), partially supported (Hypotheses 2, 3, and 5) or directly opposed (Hypothesis 4) by the evidence. On average, design partners reported significant improvement from day 2 to day 25 on all four dependent variables, confirming Hypothesis 1. Their deviations from a balanced time perspective (dBTP) became smaller by an average of 5%, indicating that through the course of the study their time perspectives became more aligned with the time perspective balance that best predicts overall wellbeing. Their physical symptoms of stress scores reduced by an average of 10%, feelings of unconditional love increased by an average of 5%, and overall wellbeing scores increased by an average of 12%. Though responses on the sliders were not part of our pre-planned hypotheses, we also noted that responses on the experimental physical, emotional and spiritual wellness sliders were significantly correlated in the expected directions with three of the four dependent variables, supporting the validity of the slider assessments. However, responses on the unconditional love measure were not correlated with responses on any of the sliders, suggesting that the unconditional love measure may capture a component of wellbeing that is non-overlapping with physical, emotional, or spiritual wellbeing (see below).

It is unlikely but possible that the changes measured during this study were due to demand characteristics, even though design partners knew our intention was to increase wellbeing. They reported liking our team, so they could have wanted to please us by showing us that they’d changed throughout the study. In support of this point, design partners who were in the recording-first condition reported consistently lower levels of physical symptoms of stress throughout the study, suggesting that scores on the physical symptoms of stress measure was the dependent variable most likely to be affected by task order – potentially resulting from disappointment at not being selected for the recording-first group. On the other hand, task order impacted improvement patterns (not simply absolute reporting levels). When task order was taken into account, participants who did the recording task first showed significantly greater reductions in their physical symptoms of stress scores compared to those who did the quote task first, a pattern that is difficult to understand based on a demand-characteristic interpretation. It is also worth noting that because the updates in the software requested by participants were provided to all participants regardless of group, the differential group effects observed here cannot be explained by these software updates. Overall, these results partially supported Hypothesis 2, that dependent variables would improve more in the recording-first versus quote-first group. This was only confirmed by significantly more improvement in the time perspective (dBTP) measure for the recording-first versus quote-first group, but for the three remaining dependent variables, significant improvements were greater in the recording-first group, and thus in the direction of supporting Hypothesis 2.

We were surprised that participants with higher amounts of self-reported childhood trauma seemed to find Time Machine particularly effective. The recurring assessments revealed significantly worse deviations from a balanced time perspective and overall wellbeing scores for design partners with many adverse childhood experiences (higher ACES scores) on the first recurring assessment, in partial confirmation of Hypothesis 3 and in opposition to a demand-characteristic hypothesis. Scores on the physical symptoms of stress and unconditional love measures, however, did not show a significant difference dependent on ACES scores at this same time point. However, while the time perspective measure remained significantly worse throughout the study for those with higher versus lower ACES scores, scores on the overall wellbeing measure were significantly worse only at the start of the study – later, individuals with greater childhood trauma essentially caught up to those with less childhood trauma with respect to overall wellbeing scores. This disassociation between the time courses of improvements in time perspective and improvements in overall wellbeing scores may potentially be explained by changes in feelings of unconditional love, as discussed below. Regardless, the significantly greater improvement on the overall wellbeing measure among design partners with higher ACES scores, combined with no significant differences in improvement on any other dependent variable, provides clear evidence *against* Hypothesis 4, which was that those with higher ACES scores would improve *less* on all dependent variables than those with lower ACES scores. When considered separately, data from both lower- and higher-ACES design partners revealed significant first-to-last improvements. However, for design partners with higher ACES scores, overall wellbeing scores increased by 16% – significantly more and twice that of the increase shown by design partners with lower ACES scores. Although any differential findings between groups with relatively lower and higher ACES scores could be due to differences in depression, anxiety, or other confounding variables – the result remains that individuals who reported greater amounts of early childhood trauma also reported, on average, twice as much improvement in the overall wellbeing measure, regardless of concomitant mental health diagnoses. To explain these population differences with response bias we would have to resort to some kind of collusion among the participants, which we think highly unlikely. Assuming no collusion, these results suggest that regardless of early childhood trauma, our design partners improved on the most critical measure of wellbeing – their overall sense of wellbeing.

Further evidence that the improvements were not entirely due to response bias arise from the results of the regression analyses and the *post-hoc* moderation and mediation analyses. The regression results indicated that both the number of optional logins and task order were related to changes in the time perspective and overall wellbeing measures, such that more optional logins and being included in the recording-first group predicted greater first-to-last improvements in time perspective and overall wellbeing scores. These results partially supported Hypothesis 5, which was that improvements in all dependent variables would increase with increases in use during the optional study period. In addition, *post-hoc* moderation analyses revealed that first-to-last improvements in time perspective and the overall wellbeing measure were moderated by the number of optional logins, significantly so for overall wellbeing scores. These results indicate either that individuals who reported greater improvements on the overall wellbeing measure were more likely to log in and do the recording task during the optional period, or that the recording task actually influenced improvements in overall wellbeing. Either way, they do not suggest response bias as a driving factor for the changes reported over time. Based on these data, and especially due to the differential task order and optional login effects, we conclude that the time travel narrative task – the recording task itself – was a driving factor in the positive shifts of the four dependent variables.

Finally, it appears that feelings of unconditional love may have played a unique role in the constellation of changes that occurred during the course of this study. First, *post-hoc* analyses revealed that improvements in feelings of unconditional love during the required portion of the study (days 2 to 14) positively predicted first-to-last improvements in both time perspective and overall wellbeing scores. Second, *post-hoc* mediation analyses revealed that, following performance of the two required tasks, greater feelings of unconditional love were likely to have positively influenced an existing relationship between a more balanced time perspective earlier in the study and a better final overall wellbeing score. Taken together with the fact that scores on the unconditional love measure were the only of the four dependent variables that did not correlate with responses on the wellness sliders, these results indicate that feelings of unconditional love may have a unique role to play in improvements in both time perspective and overall wellbeing.

These results, while promising, are limited in several important ways. First, all design partners were paid for their participation at a fairly high rate, given the amount of time involved (see Methods). For the reasons cited above it is unlikely they were being dishonest about their actual experiences, but the payment certainly motivated them to complete the study. While the results are convincing in terms of their positive impact on the participants, it is possible that it may be difficult to create a version of the technology that would be engaging enough to create enduring habits among unpaid individuals. To mitigate this limitation for future versions of Time Machine, we plan to create a more engaging interface as well as provide additional resources and opportunities for social connection among participants.

A second major limitation is that all of our effects derive from self reports. Though we do not think response bias had a major role to play here, it is also the case that people do not always have an accurate grasp on the subtler aspects of their wellbeing. Further, it is possible that a placebo effect created by the positive rapport with the design partners and the payment strategy is responsible for these results, even though the complex and ultimately coherent patterns in the results suggest that if this is a placebo effect, it is one that will be difficult to differentiate from a treatment effect. Thus future research might usefully include behavioral, implicit, or follow-up measures to further verify any effects on wellbeing.

Third, we are confident the positive quantitative results were at least partially due to the fact that our participants became well informed about the science and psychology of hope and time perspective by attending a focus group on day 1 of the study. Thus the scalability of the technology has yet to be seen. A version of Time Machine with an introductory informational and motivational video covering the essential aspects described in the focus group is being planned to mitigate this concern.

Fourth, our *post-hoc* moderation and mediation analyses are really a blunt, first-pass attempt at informing a model that accurately represents the contributions of the study parameters examined here, so we do not put much stock in the relative importance of the factors revealed by those analyses (Nathans et al., 2012). In future work with more participants, the relative contribution of each factor could be more thoroughly investigated by using an approach such as assigning each feature an importance value (e.g., “SHAP;” Lundberg and Lee, 2017).

Finally, this study only had 96 participants and was performed over 26 days – a larger study group over a longer period of time would help to further shed light on the effectiveness of self-serve time-travel narrative technology such as Time Machine.

### Research Context and Implications for Future Work

The time travel narrative approach supports the habit of creating positive personal narratives that weave the present moment into the future of an individual, for instance, by imagining that one can correspond with oneself over time by taking into account what an older, wiser version of oneself might say (Newsome, 2004). It is not surprising this approach was effective, given that even outside therapeutic or counseling contexts, brief interventions aimed at increasing the extension and adaptivity of future narrative thinking and/or goal-directed behavior resulted in fewer alcoholic drinks among alcoholics (Snider et al., 2016), reduced binge drinking in college students (Murphy et al., 2012), and increased physical activity in young adults (Hall and Fong, 2003). Further, within therapeutic or school counseling contexts, positive results have been reported from case studies in which people were coached to create a coherent and positive narrative connecting to their future selves. Examples include at-risk students (Newsome, 2004; Kress et al., 2011; Madden et al., 2011), patients with PTSD (Palgi and Ben-Ezra, 2010; Palgi et al., 2014), individuals with trauma histories (Kress et al., 2008), and those with self-injury behaviors (Hoffman et al., 2010). The present study offers the novel finding that individuals, especially those with a history of childhood trauma and neglect, could self-administer the time-travel narrative intervention in a way that effectively boosted their wellbeing within the course of a month.

To our knowledge, this is one of only three existing studies that uses a clearly defined description of unconditional love to assess feelings of unconditional love over time (Mossbridge et al., 2018, 2021), and the first study to show mediation of any effect by feelings of unconditional love. We were motivated to check for the mediation effect based on a previous study in which feelings of self-transcendence mediated the relationship between participation in a community-based wellbeing program and psychological wellbeing outcomes (Vieten et al., 2014). Self-transcendence in that study was defined as “a term used to describe (a) a desire to discover meaning in human life, (b) a growing spirituality involving both an expansion of boundaries and an increased appreciation of the present, or (c) a developmental process that forms a pathway to wisdom” (Vieten et al., 2014, p. 5, references removed). We noted the similarity between their definition of self-transcendence and our own definition of unconditional love as presented to the participants, which included a reference to everything that exists reaching its greatest possible fulfillment, a concept consistent with self-transcendence (see Methods for definition). Also, the similarity between unconditional love and self-transcendence has been pointed out previously (Goertzel et al., 2017; Staudigl, 2017). Taken together, our result showing that unconditional love mediated the relationship between time perspective and wellbeing can be considered somewhat of a replication of the results from Vieten et al. (2014).

Future Western psychological and medical clinical practitioners will likely integrate a fuller picture of human spirituality into their work, given the efficacy and growing cultural appreciation of this approach (e.g., Koenig, 2000; Sperry, 2001). At the same time, app use among both medical providers and clinical psychology patients is on the rise (Franko and Tirrell, 2012; Miralles et al., 2020). These two trends are likely to merge. However, we agree with Koenig (2000) that “Physicians should not “prescribe” religious beliefs or activities for health reasons,” but that “Physicians should acknowledge and respect the spiritual lives of patients, and always keep interventions patient-centered” (p.1708). In line with this philosophy, we think creating time travel narrative technology offering minimally instructive self-service interventions allows each individual to determine their own level of religious or spiritual involvement in the process, which can surely change over time.

Finally, it is likely that including self-transcendent concepts (like unconditional love) within the context of these technologies will support positive changes in wellbeing, at least when those concepts are also introduced in a “live” focus group prior to using such technologies. Certainly, the interpersonal relationships and caring intersubjective experiences forged in even a brief interaction can motivate change (e.g., Mitchell, 2014; Siegel, 2012; Stern, 2018). Thus it remains to be seen whether in-person or “live” clinical introduction of spiritual technologies such as these is required to produce the greatest impact on wellbeing, but we have little doubt future innovation will allow for scalable self-transcendence and its positive effects.

## Data Availability Statement

The raw data supporting the conclusions of this article will be made available by the authors, without undue reservation.

## Ethics Statement

The studies involving human participants were reviewed and approved by Institute of Noetic Sciences IRB approval: MOSJ_2020_01. The patients/participants provided their written informed consent to participate in this study.

## Author Contributions

JM, KJ, PW, AW, and MS: substantial contributions to the conception or design of the work AND the acquisition, analysis, or interpretation of data for the work, provide approval for publication of the content, and agreed to be accountable for all aspects of the work in ensuring that questions related to the accuracy or integrity of any part of the work are appropriately investigated and resolved. JM, PW, and MS: drafting the work or revising it critically for important intellectual content. All authors contributed to the article and approved the submitted version.

## Conflict of Interest

The authors declare that the research was conducted in the absence of any commercial or financial relationships that could be construed as a potential conflict of interest.

## Publisher’s Note

All claims expressed in this article are solely those of the authors and do not necessarily represent those of their affiliated organizations, or those of the publisher, the editors and the reviewers. Any product that may be evaluated in this article, or claim that may be made by its manufacturer, is not guaranteed or endorsed by the publisher.
